# Feasibility of ‘Muscle Movers’: a teacher-delivered program to support children’s participation in muscle-strengthening physical activity

**DOI:** 10.1186/s40814-025-01751-0

**Published:** 2025-12-16

**Authors:** Jordan J. Smith, Sarah G. Kennedy, Narelle Eather, Nicholas Riley, David R. Lubans

**Affiliations:** 1 Global Sport and Movement Collaborative, College of Human and Social Futures, University of Newcastle, Callaghan, NSW Australia; 2 Hunter Medical Research Institute, New Lambton Heights, NSW Australia; 3 School of Health Sciences, Western Sydney University, Kingswood, NSW Australia

**Keywords:** Strength, Physical education, Teacher, Intervention, Resistance training

## Abstract

**Background:**

Muscle-strengthening activity (MSA) is beneficial for school-aged children, but most school-based MSA interventions have been delivered by external specialists or research staff, limiting scalability. We aimed to assess the feasibility of a teacher-delivered MSA intervention for children in advance of a future efficacy trial.

**Methods:**

We conducted a single-group feasibility trial with two Stage 2 (i.e. grade 3–4) classes from one primary school in New South Wales, Australia. The 6-week *Muscle Movers* intervention included (i) enhanced PE lessons focused on foundational MSA skills (1 × 45 min/week), (ii) classroom energiser breaks (2 × 5 min/week), and (iii) active homework tasks (1 × 10 min/week). We assessed acceptability, implementation, adaptation, and practicality using survey and interview methods. We also assessed pre–post change in children’s perceived strength, upper-body muscular endurance, and lower-body muscular power. Data were analysed in SPSS (V.25) using descriptive statistics and paired-samples *t*-tests, with Cohen’s *d* as a measure of effect size.

**Results:**

Two female teachers (31 and 59 years) and 30 students (mean [SD] = 9.8 [0.6] years; 40% female) were enrolled. Acceptability was high for teachers (mean [SD] = 5.0 [0.0] out of 5) and students (mean [SD] = 4.1 [1.0] out of 5). Teachers implemented all PE lessons and more than double the intended energiser breaks (mean [SD] = 5.5 [2.1] per week). Conversely, homework task assignment (mean [SD] = 5.0 [1.4]) and completion (mean [SD] = 2.5 [0.7]) were lower than intended. Teachers reported high confidence to deliver the program and viewed it as practical and adaptable. We found a moderate increase in children’s push-up performance (mean [95%CI] = 2.2 repetitions [0.7 to 3.8]; *d* = 0.61), but no meaningful changes in perceived strength (mean [95%CI] = 0.1 units [- 0.1 to 0.4]; *d* = 0.22) or standing long jump (mean [95%CI] = - 1.4 cm [- 7.4 to 4.7]; *d* = - 0.09).

**Conclusions:**

*Muscle Movers* was feasible for classroom teachers to implement in a primary school setting. The observed improvement in students’ upper-body muscular endurance should be confirmed using an appropriately powered randomised controlled trial.

**Trial registration:**

Retrospectively registered with the Australian and New Zealand Clinical Trials Registry (ACTRN12625000703404).

**Supplementary Information:**

The online version contains supplementary material available at 10.1186/s40814-025-01751-0.

## Key messages regarding feasibility

- Muscle-strengthening activities (MSA) are largely neglected in school-based physical activity promotion. Of the trials that have focused on MSA, most have been delivered by external specialists or research staff, limiting their scalability.
- Teachers and students were highly satisfied with the ‘Muscle Movers’ program, and implementation fidelity was strong. Teachers also found the program adaptable and practical. Teachers were not affected by common barriers, including lack of time, inadequate facilities, or student motivation.
- Further evaluation of ‘Muscle Movers’ using an appropriately powered trial is warranted. A longer intervention period, additional teacher training, and strategies to support child engagement with active homework tasks may further support effectiveness and scalability.

## Background

Muscular fitness is an umbrella term encompassing three dimensions of skeletal muscle functioning, namely: (i) maximal strength (i.e. the maximum force generated against a resistance), (ii) local muscular endurance (i.e. the capacity to repeat/sustain contractions against sub-maximal resistance), and (iii) muscular power (i.e. the ability to exert force rapidly, also known as explosive strength). A large body of evidence has identified muscular fitness as an important marker of health for school-aged youth [1] showing cross-sectional [2] and longitudinal [3] associations with adiposity and other cardio-metabolic risk factors, bone mineral density, and physical self-concept. Moreover, a sufficient level of muscular fitness is necessary for successful performance across various physical activities during youth, with implications for ongoing participation [4]. This implies that muscular fitness might be an important contributor to the development of physical literacy, defined as the motivation, confidence, physical competence, knowledge and understanding to value and take responsibility for maintaining purposeful physical pursuits/activities throughout the life course [5].

Observational evidence of the benefits of muscular fitness is bolstered by decades of experimental work demonstrating the efficacy of muscle-strengthening activity (MSA; most commonly resistance training [RT]) for physical, psychological, and cognitive health among typically developing youth and diverse clinical populations [6–9]. For these reasons, international health authorities now recommend school-aged youth (5–17 years) participate in MSA on at least 3 days each week [10]. Although well supported, this advice stands in contrast to persistent beliefs among many parents and educators that MSA is either inappropriate or unnecessary for non-athletes and children [11, 12].

According to a recent meta-analysis [13], the global prevalence of ‘guideline concordant’ MSA (i.e., 3–7 days/week) among children and adolescents is 39%, although this number varies considerably across countries [14, 15]. Considering this, it is perhaps not surprising that global secular declines in muscular fitness have been reported for children as young as 9 years [16, 17]. For example, nationally representative data demonstrate that the jumping performance (i.e., lower body power) of Australian children aged 11–12 years has declined by ~ 11% since 1985 [18], and two-thirds of children in Australia’s most populous state (i.e., New South Wales [NSW]) do not demonstrate a ‘healthy’ level of muscular fitness [19]. Taken together, these data imply children are participating in less MSA than they did in previous generations and less than recommended for optimal health and development. MSA participation differs according to several individual and social factors (e.g. gender, weight status, physical activity level, family support) [13], and so there is a need for equitable interventions that can reach diverse groups of young people to redress health disparities.

Schools have long been used as a setting for physical activity promotion and are generally considered equitable and cost-effective compared with other intervention settings [20]. Although many school-based interventions have targeted students’ (predominantly aerobic) moderate-to-vigorous intensity physical activity [21], far fewer have focused on MSA. A meta-analysis published in 2022 identified 17 published studies (*N* = 1653 participants) examining the efficacy of MSA-focused school-based interventions for children (<13 years), and reported moderate pooled effects for local muscular endurance and strength/power [22]. While providing useful empirical support for the efficacy of MSA interventions in the school setting, information on intervention characteristics (e.g. delivery agent, resource provision) was limited. Closer inspection of the included studies shows that, while usually leveraging the school Physical Education (PE) period, interventions were rarely delivered by ‘generalist’ classroom teachers. Instead, most interventions were delivered by external providers, specialist PE teachers, or trained research staff [22]. These delivery models are either impractical or cost-prohibitive for implementation at scale.

Classroom teachers are often responsible for delivering traditional aerobic and game/sport-based physical activity interventions in schools [23], but many teachers consider MSA to be more complex or risky than familiar aerobic activities and games/sports [24]. Indeed, even specialist PE teachers have expressed a lack of experience and confidence to deliver MSA [24], and this group is far more prepared than generalist teachers given the limited PE-related coursework within most pre-service teacher training programs. Generalist teachers may also experience additional logistical (i.e. lack of space/equipment) and attitudinal barriers (i.e. personal beliefs that MSA is inappropriate), which could hamper the adoption and implementation of MSA-focused programs [25]. This emphasises the need for thoughtful intervention design when promoting ‘teacher-delivered’ MSA in schools (particularly primary/elementary schools), as decisions on intervention content, complexity, space/equipment needs, requisite knowledge/training, and resourcing may all be critical. That said, even a logistically feasible and ostensibly attractive MSA intervention may fail to be adopted, properly implemented or sustained if it is viewed as competing for time with more ‘necessary’ learning (e.g. literacy and numeracy). Clearly communicating exactly ‘how’ such programs align to outcomes and content within mandatory PE curricula may thus be important for obtaining ‘buy in’ from various stakeholders (i.e. from departments of education through to individual teachers).

Prior to the commitment of resources for testing efficacy/effectiveness, it is important to first establish whether an intervention is feasible within the target setting. This is particularly relevant where there is an absence of similar examples within the literature. To our knowledge, no prior trial has evaluated a curriculum-aligned MSA intervention for primary school children delivered by generalist teachers. Therefore, the aim of our study was to evaluate the feasibility of *Muscle Movers*, a school-based intervention to support children’s skill development and participation in MSA consistent with international recommendations. The findings from this trial will be used to identify program components in need of further refinement and to determine whether progressing to a fully powered efficacy trial (i.e. cluster randomised controlled trial [RCT]) is warranted.

## Methods

### Study design, participants, and setting

The conduct and reporting of this study comply with the Consolidated Standards for Reporting Trials (CONSORT) extension for randomised pilot and feasibility trials [26]. Approval for the study was obtained in October 2022 from the Human Research Ethics Committee of the University of Newcastle (H-2022–0273) and the Catholic Schools Office of the Diocese of Maitland-Newcastle. The intervention was evaluated using a single-group experimental design (pre–post assessments) within one Catholic primary school located in NSW, Australia. Informed consent was obtained from the school Principal, participating teachers, and parents/carers of all students prior to their enrolment in the trial.

Although evidence supports the expectation of a moderate effect on muscular fitness with this population and setting [22], we adopted a conservative assumption of a small treatment effect (Cohen’s *d*∼0.1–0.3). According to Whitehead and colleagues [27], under this effect size assumption, a sample size of 25 participants per treatment arm is sufficient to estimate the variability needed to determine the sample size for a definitive trial (with 90% power and α = 0.05). Our planned sample of 30 participants therefore provides comparable or greater precision for estimating variability in this single-arm feasibility study, as well as additional buffer to accommodate up to 20% attrition. Two Stage 2 teachers (i.e. Grades 3–4; ~9–10 years old) were recruited using a convenience sampling approach to deliver the *Muscle Movers* program with their classes over 6 weeks during school term 4, 2022 (November–December). Informed consent was received from the parents of almost all students in the two classes (*N* = 30; consent rate = 93.8%).

Baseline and post-test assessments occurred on school premises within the same school term directly prior to and following the intervention period by two unblinded researchers with postgraduate PE qualifications. Self-report student measures were collected using hard copy surveys in the classroom, whereas fitness measures were conducted with small groups in a separate room. Self-report teacher measures were collected using online surveys at the same two time points. Participating teachers received a small gratuity ($100AUD) at the conclusion of the trial in recognition of their contributions to the study.

### Theoretical background

*Muscle Movers* was informed by Beets and colleagues’ theory of expanded, extended, and enhanced opportunities [28], which is a pragmatic theory that emphasises the importance of regular structured opportunities for young people to be physically active. According to this theory, interventions can increase young people's physical activity by extending (increasing the duration) and enhancing (increasing the efficiency of) existing opportunities, or by expanding (creating new) opportunities for physical activity participation. Combining these strategies is expected to achieve greater improvements than might occur through any single strategy in isolation. As such, *Muscle Movers* targeted both expansion (energiser breaks, active homework) and enhancement (PE program) approaches to increase children’s MSA participation towards global physical activity recommendations [10].

### Intervention development

We used the conceptual framework by Morgan and colleagues [29] to guide intervention development. This framework outlines a targeted approach to intervention design that in the first instance considers relevant socio-cultural characteristics of end users (i.e. children and teachers) and uses these insights to inform decisions relating to four core intervention components (i.e. content, format, facilitator, pedagogy). A detailed summary of the intervention, aligned to the components of Morgan and colleagues’ conceptual model, is provided in Additional file 1. The program and supporting resources were also developed with reference to the SAAFE framework [30], which outlines five evidence-based principles (Supportive, Active, Autonomous, Fair, Enjoyable) intended to guide the delivery of organised physical activity sessions for school-aged youth. Guided largely by self-determination theory [31], the SAAFE principles have been employed across a number of school-based programs to increase physical activity and support physical literacy whilst satisfying students’ basic psychological needs [32–34].

### Intervention description

An overview of the *Muscle Movers* intervention components is provided in Table 1. *Muscle Movers* included three distinct components intended to provide children with opportunities to participate in MSA throughout the school week: (i) a curriculum-aligned PE program; (ii) classroom energiser breaks; and (iii) active homework tasks. These were supported by a classroom resource package developed by the research team and provided free of charge to participating teachers. The package included a teacher handbook, USB drive/Cloud drive access (for electronic resources), PE equipment pack, exercise skill cards, classroom wall charts, and student handbooks. Given the age/experience of the target population, the program aimed to build competence across four foundational bodyweight RT movement skills, considered to form the basis for more advanced RT exercises.

**Table 1:** Intervention components

Component (TEO strategy)	Exercise programming information	Supporting resources
PE program (Enhancement)	WHAT: 1 × 45 min/week for 6 weeks. Beginner (assumed starting point) body weight resistance exercises (i.e. squat, lunge, plank, push-up) taught/practiced in isolation and then incorporated within minor games using fitness infusion strategy. No specific reps/sets prescribed. Generic exercises, some differentiation possible via progression/regression information on skill cards WHO: Delivered by classroom teachers with no prior exercise-related qualifications, apart from Physical Education coursework within pre-service teaching degree. First lesson delivered by lead researcher to familiarise teachers with lesson structure, and model effective instruction/pedagogy HOW: Children participate as a group with peers from their class. Lessons supervised/instructed by classroom teacher, and designed/delivered in accordance with the SAAFE (supportive, active, autonomous, fair, enjoyable) principles. Principles support motivation by satisfying children’s basic psychological needs for autonomy, competence, and relatedness. Children recognised for demonstrating prosocial interpersonal and self-management skills WHERE: Outdoors in school playground spaces	• Teacher handbook • Master Move skill cards • PE equipment pack • Wall charts
Energiser breaks (expansion)	WHAT: 2 × 5 min/week for 6 weeks. Beginner (assumed starting point) and intermediate body weight resistance and aerobic exercises. 5 × 20 s intervals interspersed with rest. No specific sets/reps prescribed. Generic exercises, no differentiation (unless to address precluding injury) WHO: Facilitated by classroom teachers, with no prior exercise-related qualifications, apart from Physical Education coursework within pre-service teaching degree. No training provided to teachers HOW: Children participate as a group with peers from their class. Energisers supervised by classroom teacher. PPT files with themed energisers projected on Smartboard. Children follow prompts and complete exercises as illustrated on screen WHERE: Inside school classroom	• USB/cloud drive with electronic files
Active homework (expansion)	WHAT: 1 × 10 min/week for 6 weeks. Beginner (assumed starting point) body weight resistance exercises incorporated into at-home challenges presented within student handbook (i.e. squat, lunge, plank, push-up). No specific sets/reps prescribed. Generic exercises, no differentiation WHO: Self-directed by children, with support from parent/guardian. Children instructed on exercises at school. No training provided to parents/guardians HOW: Children participate individually or with family members. Activity challenges outlined within student handbook. Children follow instructions and complete relevant activity WHERE: Within family home	• Student handbook

**Fig. 1:**
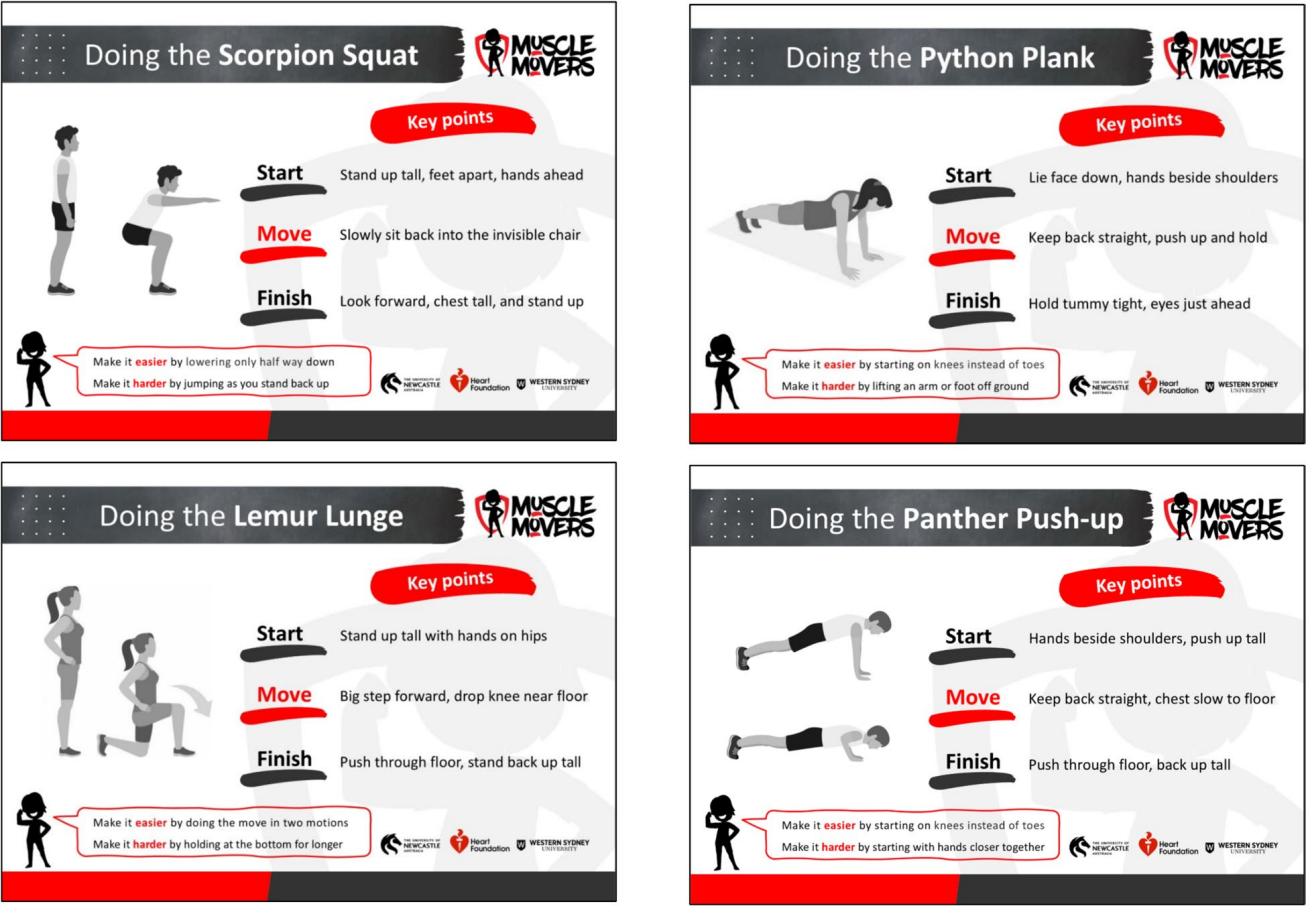
Master Move skill cards

The four movement skills, referred to within the program as the *Master Moves*, were given animal names to make them more engaging and memorable for children: (i) *Scorpion Squat*, (ii) *Lemur Lunge*, (iii) *Python Plank*, and (iv) *Panther Push-up*. The skills were outlined within the teacher handbook and on hard copy cards for teachers and students to use during PE lessons. The cards (see Fig. 1) provided an illustration of the skill, simple technique cues organised across the three phases of the movement (*start*, *move*, *finish*), and suggested modifications for differentiation (i.e. to increase/reduce the degree of challenge for children with differing abilities).

Physical literacy domains were addressed through the intervention in the following ways: (i) competence was developed through explicit instruction and practice of the four *Master Moves* with progression/regression options; (ii) confidence and motivation were fostered through delivery consistent with the SAAFE principles and by embedding skills into enjoyable minor games, and (iii) knowledge/understanding was reinforced through curriculum-linked teacher resources that explained the relevance of MSA to children’s health and future activity participation. Although we intended to support children’s physical literacy in these ways, it is important to note these domains were not measured empirically in this study.

#### PE program

Detailed information regarding the structure and content of each PE lesson is provided in Additional file 1, and an example lesson plan is provided in Additional file 2. In brief, teachers received a semi-prescriptive program comprised of 6 weeks of sequential PE lesson plans (1 × 45 min/week) designed by the research team. Each lesson was divided into three segments: (i) *Start Strong* (i.e. engaging warm-up activity), (ii) *Main Muscles* (i.e. the main body of the lesson involving explicit MSA skill instruction and application within a game-based context), and (iii) *Muscle-Up* (i.e. optional extension activity to add variety). Teachers were asked to deliver the *Main Muscles* segment as prescribed each week but were free to exchange, repeat, or re-order any activities aligned with the other lesson segments to suit their needs/preferences. The program was linked to stage-appropriate curriculum outcomes and presented within the teacher handbook.

To familiarise teachers with the PE lesson format, corresponding resources, and to model correct instruction of the four *Master Moves*, the first PE lesson was delivered by a member of the research team while the teacher observed. In addition, the lead researcher attended the school for one observation visit (week 3 of 6) to assess implementation fidelity and to provide advice/support to teachers if needed. Two hard copy wall posters for the classroom were provided to teachers to support PE lesson outcomes. The *Managers* wall chart supported teachers in allocating student helpers for transport, set-up, and pack-up of PE equipment. The *Motivators* wall chart supported teachers in reinforcing and recognising students’ ‘self-management’ and ‘interpersonal’ skills, which, along with ‘movement skills,’ represent the three skill domains outlined in the NSW K-10 *Personal Development, Health and Physical Education (PDHPE)* syllabus [35].

#### Classroom energiser breaks

Energiser breaks were developed by the research team to provide teachers with a scaffold for delivering additional MSA opportunities during otherwise sedentary periods of the school day. Teachers were encouraged to deliver two energiser breaks each week on days PE lessons were not delivered. Energiser breaks were developed using Microsoft PowerPoint with pre-programmed automatic transitions and provided to teachers on a USB drive (and online via a cloud-based service). Twelve unique energiser breaks were developed, organised into two formats to ensure sufficient variety and prevent boredom: (i) *would you rather* breaks used distinct themes (e.g. food, occupations), with students choosing one of two options and performing the matching exercise shown on screen for 20 s; (ii) *Rock, paper, scissors* breaks followed the classic game; students acted out their choice, then compared it with the option displayed on screen to see if they won, lost, or drew the round. They then performed the matching exercise for 20 s. Each energiser break included five rounds and lasted just under 5 min.

#### Active homework tasks

To provide an additional opportunity for MSA beyond the school day, the research team developed a student handbook with six active homework tasks designed to be engaging and enjoyable for children. Teachers were instructed to assign one active homework task each week from the handbook. Tasks were designed to be brief (approx. 10 min each), age-appropriate, to promote family involvement, and to enable additional practice of the four *Master Moves*.

### Feasibility measures

#### Quantitative evaluation

We used the framework outlined by Bowen and colleagues [36] to assess feasibility across the following four domains: (i) acceptability, (ii) implementation, (iii) adaptation, (iv) practicality. As the trial did not include a control group, preliminary efficacy could not be ascertained. However, we included pre–post measures for several student outcomes to determine whether changes were in the hypothesised direction. A description of the feasibility domains and the list of corresponding measures is provided in Table 2. A range of quantitative feasibility measures was collected using an online survey for teachers and a hard copy survey for students. Students responded to a single item at baseline regarding their frequency of MSA over the past week (possible range = 0 to 7 days per week). Illustrative examples included (i) strength-based exercises (i.e. push-ups, sit-ups, lifting weights), (ii) climbing and jumping activities, and (iii) yoga or gymnastics. Teachers also rated each individual PE lesson activity (range: 1 = poor to 5 = excellent) within their teacher handbook, and these data were collected at the post-test time point. The handbook included space for teachers to record adverse events (e.g. physical injury) occurring as a result of the intervention, which was used to determine potential harms.

**Table 2:** Feasibility domains assessed in the current study

Domain	Description	Student measures	Teacher measures
*Acceptability*	The extent to which the program is considered suitable, satisfying, or attractive to program participants	• Overall enjoyment of program • Enjoyment of PE lessons • Enjoyment of energiser breaks • Enjoyment of homework tasks	• Program satisfaction • Likelihood of recommending to others • Intention to sustain delivery • Perceived benefits for students • Colleagues’ support for program • Ratings of PE lesson activities
*Implementation*	The extent, likelihood, and manner in which the program can be fully implemented as planned		• Number of PE lessons delivered • Number of energiser breaks delivered • Number of home tasks assigned and completed • Perceived ease of implementation • Confidence with program delivery • Difficulty motivating students
*Adaptation*	The extent to which an existing program can be adapted to fit the needs of different populations		• Perceived adaptability of program
*Practicality*	The extent to which the program can be delivered using existing resources or with limited resources, and without outside intervention		• Use of program resources • Perceived quality of program resources • Suitability of school facilities

#### Qualitative evaluation

After the conclusion of the intervention, semi-structured interviews were conducted by the lead author (male, PhD, Senior Lecturer), who had no prior relationship with participants but is experienced in school-based physical activity research. He was mindful of his professional background and potential influence and emphasised the value of teachers’ perspectives. An interview guide was developed with reference to Bowen and colleagues’ [36] feasibility domains. Interviews were conducted face-to-face at the school, lasted ~ 20 min, and were audio-recorded with consent. No other persons were present, and no field notes were taken. Interviews were transcribed verbatim by a research assistant, and transcripts were checked for accuracy by the lead author. Rather than a formal thematic analysis, a descriptive content approach was applied: transcripts were read by the lead author and examined against the pre-specified feasibility domains, and salient quotes were extracted to illustrate key findings. Given the small, pre-determined sample (*n* = 2 teachers), data saturation was not applicable. Transcripts were not returned to participants, and member checking was not conducted, as the qualitative component was designed only to provide supplementary insight into program feasibility. Illustrative quotations are presented in the Results and labelled by participant (e.g. Grade 3 teacher, 31 years).

### Teacher measures

#### Confidence to teach muscle-strengthening activities

Assessed using a scale originally developed to explore teachers’ experiences delivering PE programs [37], but adapted to apply to teaching MSA. After reading a definition of MSA, teachers responded using a 5-point Likert scale (1 = strongly disagree, 5 = strongly agree) to two items assessing their confidence to teach MSA generally (e.g. “Overall, I feel confident to teach muscle-strengthening activities to children”), and bodyweight exercises specifically (e.g. “I feel confident to teach exercises such as push-ups and sit-ups to children”).

#### Perceived barriers to teaching muscle-strengthening activities

Assessed using a scale originally developed to assess teachers’ perceptions of barriers to delivering PE but adapted to apply to MSA [38]. Teachers were asked to indicate “the degree to which the following act as barriers or inhibit your capacity to deliver muscle-strengthening activities in your school” and responded using a 6-point scale (1 = no barrier or does not inhibit; 4 = moderate barrier; 6 = a major barrier or strongly inhibits). The scale was comprised of 12 items clustered into two broad categories [39]. A contextual barrier composite score was calculated as the mean of responses to (i) inadequate facilities/space, (ii) class size too big, (iii) lack of time, (iv) inadequate equipment, (v) litigation concerns, and (vi) lack of money budgeted to programs. An *interpersonal* barriers score was calculated as the mean of responses to (i) low levels of teaching confidence, (ii) poor level of staff support provided, (iii) low levels of personal interest and enthusiasm, (iv) negative executive attitudes, (v) lack of departmental assistance/professional learning, and (vi) negative student attitudes.

### Student outcome measures

#### Perceived strength

Assessed using the perceived strength subscale of Marsh’s Physical Self-Description Questionnaire [40]. Students responded to six items (e.g. “I am a physically strong person”) using a 6-point scale to indicate how true each statement was for them (1 = false, 6 = true). Given the age of the study sample (i.e. 9–10 years), response options were depicted using a spectrum of ‘smiling/frowning faces’ to aid interpretation. The internal consistency of scale items among the study sample at baseline was high (Cronbach α = 0.88).

#### Upper body muscular endurance

Assessed using the 90° push-up test [41], which is a widely used field-based test of upper body muscular endurance. Following an explanation and demonstration from the assessor, participants completed as many push-ups as possible on their toes or knees (self-selected choice) in time with a cadence set at 40 bpm. Participants’ choice of toes or knees was recorded at baseline, and the same option was completed at post-test.

#### Lower body muscular power

Assessed using the standing long jump test [42], which is considered among the most valid and reliable field-based tests of muscular fitness for use with school-aged youth [43]. After an explanation and demonstration from the assessor, participants stood on a standing long jump mat (Toei Light®) with their toes just behind a line marked at 0 cm and jumped forward as far as possible. Participants completed two test trials, and the greater distance was recorded as the result.

### Statistical analysis

Data for acceptability, implementation, adaptation, and practicality were summarised using descriptive statistics (i.e. mean and standard deviation or counts and percentage, as appropriate). Although two classes of students were recruited, they completed the main intervention component (i.e. PE lessons) together and so were treated as a single group for analyses of pre–post change. Student outcomes were analysed using paired-samples *t-*tests, which were used to calculate mean change scores and their corresponding 95% confidence intervals. The study was not powered to detect statistically significant effects; therefore, Cohen’s *d* is reported as a measure of effect size, with values of 0.2, 0.5, and 0.8 considered small, medium, and large effects, respectively [44].

## Results

The flow of participants through the study is illustrated in Additional file 3. Two female generalist teachers (31 and 59 years old) and 30 students (mean [SD] age = 9.8 [0.6] years, 40.0% female) were enrolled at baseline. Both teachers and all students were born in Australia and spoke English as their primary language at home. The teachers had 9 and 37 years of teaching experience, respectively, and neither had attained separate health/fitness qualifications. At baseline, teachers’ confidence to teach MSA generally (mean [SD] = 3.0 [1.4] out of 5) and bodyweight exercises specifically (mean [SD] = 3.5 [0.7] out of 5) was neutral. Of the 12 potential barriers to delivering MSA, only two were identified as having at least a ‘moderate’ impact (i.e. mean score ≥ 4 out of 6); ‘lack of departmental assistance/professional learning’ (mean [SD] = 5.0 [0.0]) and ‘lack of money budgeted to programs’ (mean [SD] = 4.5 [0.7]). Teachers generally perceived ‘contextual’ barriers (mean [SD] = 2.6 [0.4]) as more salient than ‘interpersonal’ barriers (mean [SD] = 1.9 [0.4]). Based on students’ self-report, 85.2% satisfied the MSA recommendation of ≥3 days/week, with 3 days being the modal response (*n* = 7; 25.9%). No students indicated 0 days of MSA.

### Feasibility evaluation

No adverse events were reported by teachers, and none of the study participants withdrew prior to the conclusion of the trial. A summary of results for teacher-reported feasibility measures is provided in Table 3 and described in detail below.

**Table 3:** Summary of feasibility evaluation for teachers (*N* = 2)

Feasibility domain	Survey items/measures	Result^a^
Acceptability	Overall satisfaction with program (/5)	5.0 (0.0)
	Would recommend program to others (/5)	5.0 (0.0)
	Intend to sustain delivery, *n* (%) ‘yes’	2 (100)
	Likely to deliver PE lessons again (/11)	11.0 (0.0)
	Likely to deliver energiser breaks again (/11)	11.0 (0.0)
	Likely to deliver home tasks again (/11)	9.5 (2.1)
	Perceived benefits to students’ physical health (/5)	5.0 (0.0)
	Perceived benefits to students’ mental health (/5)	5.0 (0.0)
	Other teachers supportive of program (/5)	5.0 (0.0)
	School executive supportive of program (/5)	5.0 (0.0)
	Mean rating for *Start Strong* lesson activities (/5)	4.9 (0.3)
	Mean rating for *Main Muscles* lesson activities (/5)	4.5 (0.5)
	Mean rating for *Muscle-Up* lesson activities (/5)	4.4 (0.7)
Implementation	Number of PE lessons delivered overall	6.0 (0.0)
	Number of energiser breaks delivered per week	5.5 (2.1)
	Number of home tasks assigned overall	5.0 (1.4)
	Number of home tasks students completed	2.5 (0.7)
	Perceived ease of implementation (/5)	5.0 (0.0)
	Confidence with program delivery (/5)	5.0 (0.0)
	Difficult finding time to implement (/5)	1.0 (0.0)
	Difficult to motivate students for PE lessons (/5)	1.0 (0.0)
	Difficult to motivate students for energisers (/5)	1.0 (0.0)
	Difficult to motivate students for home tasks (/5)	3.0 (1.4)
Adaptation	Perceived adaptability of program (/5)	5.0 (0.0)
Practicality	Perceived quality of program resources (/5)	5.0 (0.0)
	Perceived suitability of school facilities (/5)	5.0 (0.0)
	Used program resources for most PE lessons (/5)	5.0 (0.0)
	Used *Managers* wall chart, *n* (%) ‘yes’	1 (50.0)
	Used *Motivators* wall chart, *n* (%) ‘yes’	2 (100)

^a^Values are mean and standard deviation unless otherwise specified

#### Acceptability

Overall, teachers and students found *Muscle Movers* highly acceptable. Teachers reported high overall satisfaction, would recommend the program to others, believed it benefited their students’ physical and mental health, and felt the program was supported by colleagues. Teachers also reported strong intentions to sustain delivery in future, particularly the PE and energiser break components. Teacher ratings across all PE activities were high (mean [SD] = 4.6 [0.5] out of 5), suggesting the selected activities were appropriate. Students (*n* = 28 [response rate = 93.3%]) similarly reported high overall enjoyment of the program (mean [SD] = 4.1 [1.0] out of 5), with the highest satisfaction for the energiser breaks (mean [SD] = 4.9 [0.3] out of 5), followed by PE lessons (mean [SD] = 4.1 [0.9] out of 5), and active homework (mean [SD] = 3.5 [1.2] out of 5). Quantitative results were supported by qualitative responses from teachers, who reiterated student engagement with the PE program, even among those not usually engaged in PE:

“It was a lot of fun…the kids really were enjoying the new activities, and it was moving fast but it was different things they have never done, and they really liked going back and doing it…and each week if I didn’t do those energisers it was strife…so it was really, really good.” (Grade 4 teacher, 59 years)

“[Student] would have been the person to fake an injured leg prior, but she didn’t with this.” (Grade 3 teacher, 31 years)

The main constructive feedback from teachers related to the desire for more PE lessons and energiser break resources, or the option to use a template to create energisers of their own tailored to students’ interests:

“Yeah, like a whole term’s worth [of PE lessons] would be awesome. Instead of just six.” (Grade 3 teacher, 31 years)

“Yes, more of them [energiser breaks]. Or maybe even a blank where you have the exercise and teachers can put things inside the picture? That will allow us to target kids that might not really like moving [be]cause they will show interest in them” (Grade 4 teacher, 59 years)

#### Implementation

Implementation fidelity was high for the PE lessons (6 out of 6 lessons delivered) and energiser breaks (mean [SD] = 5.5 [2.1] out of 2 breaks/week delivered) but lower for the active homework component. Teachers assigned slightly fewer tasks than intended (mean [SD] = 5.0 [1.4] out of 6) and indicated only half of students typically completed the assigned tasks (mean [SD] = 2.5 [0.7]). Teachers reported the program was easy to implement and indicated high confidence with program delivery. This was further supported by teachers’ post-intervention responses to the scale items, with mean (SD) increases from baseline of 2.0 (1.4) and 1.5 (0.7) for confidence to teach MSA and bodyweight exercises, respectively. Teachers also indicated no difficulty finding time for the program nor with motivating students to participate in PE lessons or energiser breaks. Some difficulty motivating students to complete homework tasks was apparent. Additional insights regarding facilitators to implementation emerged from the teacher interviews, with teachers identifying the value of the pre-prepared structure and supporting resources:

“It was really good [be]cause it was all ready for the teacher… that’s definitely a barrier… they don’t have the knowledge of how to structure it.” (Grade 3 teacher, 31 years)

“…it was great that I had all the equipment, so I didn’t have to hunt for it and I didn’t have to fight the fact that it had gone out on to the playground at recess and lunch and got destroyed by other kids.” (Grade 4 teacher, 59 years)

Regarding the energiser breaks, one teacher identified the gender-neutral themes, music, and movements as a facilitator to implementation and contributor to student engagement:

“Everyone was easier to get involved because there wasn’t that gender barrier which always put me off doing brain breaks in the classroom...because I was always battling something, and I cannot cope with that.” (Grade 4 teacher, 59 years)

Teachers noted the variability in student engagement with the active homework tasks, which may in part have been due to the timing of program delivery, but perhaps also due to insufficient guidance provided within the teacher handbook:

“We started off with high participation but by the end it faded out…and I think that was more involved with not the actual activities but the fact that the school came to the end of the year and I was away for a couple of the days, and the kids are winding down and we’ve got Christmas concert practice and all that…I think that impacted it.” (Grade 4 teacher, 59 years)

“Yeah, that was probably the most challenging…So I had a couple of kids, like I handed it out and like they brought it back the next day and they had done all of them… so I probably needed to explain it a bit better, you know ‘when’ they do it.” (Grade 3 teacher, 31 years)

Despite the challenges, teachers identified some successes with the homework task component, including for students with additional learning needs:

“I actually had comments from parents saying ‘that was great, they were so keen to do them as soon as they got home’…I noticed that it was kids with ADHD that were like ‘I love these’ and the parents as well, it got them something to do that was kind of fun homework for them rather than reading.” (Grade 3 teacher, 31 years)

#### Adaptation and practicality

Teachers perceived the program to be highly adaptable and practical, with strong ratings for the perceived quality and usage of program resources, as well as the perceived suitability of available school facilities. Of the most salient barriers to teaching MSA identified at baseline, all were lower at post-intervention; ‘lack of departmental assistance/professional learning’ (mean [SD] = 2.0 [1.4]), ‘lack of money budgeted to programs’ (mean [SD] = 1.0 [0.0]). Moreover, the composite score for ‘contextual’ barriers was 50% lower (mean [SD] = 1.3 [0.4]) compared with baseline, and at post-intervention no specific barrier had a mean score indicating at least a ‘moderate’ impact on teaching MSA. Teachers’ qualitative responses reinforced the quantitative findings for adaptability, with one teacher noting the content of the exercise cards supported her to adapt her delivery for a student with disability:

“We have a little boy with cerebral palsy, so we adapted it for him…He just needs some extra support with that…But those ‘too easy’, ‘too hard’ things [on the exercise cards] basically did that for us.” (Grade 3 teacher, 31 years)

“I adapted them [PE lessons] all the time because if it didn’t work there was always another way of adapting it to make it work.” (Grade 4 teacher, 59 years)

### Pre–post change in student outcomes

A summary of findings for student outcomes is presented in Table 4. We observed a moderate improvement in push-ups performance (mean [95%CI] = 2.2 [0.7, 3.8] repetitions; *d* = 0.61). No meaningful changes were found for students’ perceived strength or standing long jump performance.

**Table 4:** Change in student outcomes

Outcomes	*N*	Baseline Mean (SD)	Post-test Mean (SD)	Mean change (95%CI)	*d*
Perceived strength, units^a^	25	5.0 (0.9)	5.1 (0.9)	0.1 (- 0.1, 0.4)	0.22
Push-ups, repetitions^b^	25	5.5 (6.7)	7.7 (7.8)	2.2 (0.7, 3.8)	0.61
Standing long jump, cm^c^	26	129.9 (17.8)	128.5 (17.0)	- 1.4 (- 7.4, 4.7)	- 0.09

*CI* confidence intervals, *MSA* muscle-strengthening activity, *SD* standard deviation

^a^Five students did not provide complete data for perceived strength

^b^Three students were absent and two students had a precluding injury for the push-up test

^c^Three students were absent and one had a precluding injury for the standing long jump test

## Discussion

The primary aim of our study was to evaluate the feasibility of the teacher-delivered *Muscle Movers* intervention for primary school children. Our findings suggest the program was well-received by both teachers and students, and for teachers, the intervention was viewed as practical and adaptable, leading to strong implementation fidelity. Although we saw no demonstrable change in students’ perceived strength or lower body power, we did observe a potentially meaningful improvement in upper-body muscular endurance.

Encouragingly, both teachers and students indicated high satisfaction with *Muscle Movers*, including for the PE component. This is important, given the PE program accounted for the most time and was where children received explicit instruction on MSA-related skills. Both teachers delivered all intended PE lessons, rated individual PE activities highly, and appeared unaffected by common barriers to the delivery of school physical activity programs (i.e. lack of time, low self-efficacy, inadequate facilities, low student motivation) [45]. Although not tested statistically, it was encouraging that teachers’ self-reported confidence to teach MSA improved across the study period. This is despite teachers receiving no formal training, apart from observing the first PE lesson delivered by the lead researcher.

Our positive findings for teacher confidence may be due to the consideration given to PE lesson content and supporting resources, which were designed to address common challenges experienced by generalist teachers [45]. For example, we selected familiar minor games that are enjoyable for children with diverse abilities, easy to organise and explain, and adaptable to different school environments. We also provided curriculum-aligned lesson plans within the teacher handbook to scaffold implementation and reduce teachers’ cognitive load and planning burden. This approach was semi-prescriptive, in that we expected the *Main Muscles* lesson segment to be delivered as prescribed, but teachers were free to use, exclude, exchange, or repeat prescribed activities from the *Start Strong* and *Muscle-Up* lesson segments based on their needs (i.e. time, space) and students’ preferences. These and other design choices appear to have been effective, with both teachers viewing the program as highly adaptable, the resources as high quality, and available school facilities as suitable.

When designing the intervention, we recognised the implementation challenges that emerge when interventions are too rigid and cannot be adapted to local contextual factors [46]. Deviations from intended intervention protocols (i.e. ‘program drift’) are one explanation for the reduction in effect size observed as physical activity interventions are scaled up [47], a phenomenon known as the ‘scale-up penalty’ or ‘voltage drop’ [48]. In addition, pilot trials are often limited by ‘generalisability biases’, defined as the degree to which features of the intervention and sample in the pilot study are *not* scalable or generalisable to the next stage of testing in a larger efficacy/effectiveness trial [49]. Importantly, voltage drop is greatest between pilot and efficacy/effectiveness trials of (ostensibly) the same intervention when generalisability biases are present at the pilot stage. This is particularly stark in the case of changes to the delivery agent, intervention duration, or level of implementation support, all of which are associated with reductions in the standardised mean difference for the primary outcome of > 0.32 [49]. *Muscle Movers* was designed with scalability in mind, avoiding the risk of generalisability biases common among many past behavioural interventions for children, and allowing for teacher adaptation to address unforeseeable local constraints [50].

An interesting finding was the substantially higher than expected delivery of classroom energiser breaks. Teachers were asked to facilitate two energisers each week. In practice, teachers delivered more than twice this number, with one of the teachers at times facilitating 2–3 breaks per day. Teachers’ strong engagement with this component is consistent with a large body of research demonstrating the popularity of energisers [51, 52], as well as their benefits for student behaviour and academic performance [53]. This said, there are barriers to implementing energiser breaks, and the characteristics of specific formats might influence their adoption by teachers [51]. Our data suggest the breaks included within *Muscle Movers* satisfied several criteria considered important by teachers, namely low threat to classroom control, ease of implementation, and perceived enjoyment for students [51]. The two formats of energisers we developed ensured variety, offered opportunities for student choice, and incorporated age-appropriate themes, music, and humour to promote enjoyment. Additionally, exercise intervals were based on time rather than a fixed number of repetitions to prevent unhelpful peer comparisons of fitness (i.e. children completing reps faster vs slower), which could undermine student self-efficacy and motivation [54].

The active homework component of *Muscle Movers* showed mixed success and emerged as an area requiring further refinement. While intended to extend MSA opportunities beyond the school setting, homework task completion was lower than anticipated, with 83% of intended tasks assigned by teachers and only half of students engaging with assigned tasks each week. Teachers identified end-of-year timing, competing school demands, and a lack of clarity around expectations as possible barriers to their implementation of this component. Additional guidance and structure may be necessary to support teachers’ implementation and students’ adherence. Despite the challenges, teachers also shared successes, particularly for students with additional learning needs (e.g. those with attention deficit hyperactivity disorder) who found the tasks engaging and were motivated to involve family members.

Our promising findings support the potential value of active homework, but prior evidence casts doubt on this position. For example, a 2024 review of homework interventions found benefits only for light-intensity activity [55]. Similarly, a 2025 meta-analysis of homework interventions found no improvement in device-measured physical activity (SMD [95%CI] = 0.05 [- 0.12, 0.22]), despite finding benefits for sedentary and sleep time [56]. These reports focus on intervention effects and do not explore the ‘reasons’ for the null findings. However, the lack of teacher oversight inherent in any ‘out-of-school time’ component is likely to result in poorer adherence relative to components within a teacher’s direct control at school. Competing parental priorities, variable household routines, and distractions in the home environment (e.g. recreational screen devices) may always present challenges to adherence for active homework. Despite prior evidence seeming to argue against this strategy, it may be premature to exclude it from a future iteration of the *Muscle Movers* program, given prior studies have not focused on MSA and considering the positive reports from teachers regarding neurodiverse students. However, more thought on how to support teacher implementation and increase student adherence is needed. Providing clearer instructions for teachers and direct communication with parents/guardians may be useful.

It was unexpected that most students (85%) reported engaging in at least 3 days of MSA in the past week, thereby meeting the MSA component within international guidelines for school-aged youth [10]. Indeed, around 45% reported participation on 5–7 days, which seems implausible. At face value, these results appear to undermine the need for a program such as *Muscle Movers*. However, we interpret this finding with caution given concerns about the validity of self-reported MSA in this age group (9–10 years). There is currently no universally accepted method for assessing MSA among children. Most previous studies rely on a single-item question about the weekly frequency of traditional strength-based exercises (e.g. push-ups, sit-ups, or weightlifting) [13]. This may be appropriate for adolescents but less so for younger children, whose MSA is more likely to occur through unstructured play. In this study, we sought to make the measure more developmentally appropriate by expanding the examples to also include climbing and jumping activities, yoga, and gymnastics. While this approach may have captured a broader range of relevant behaviours, it might also have inflated estimates by including common activities with variable intensity and muscle-loading characteristics. Whether this adaptation improved measurement validity remains uncertain, highlighting the need for more research on age-appropriate MSA assessment. Future measures should aim to capture not only the frequency of MSA performed but also the type and context (i.e. physical activity domain, setting) as well as other aspects of the dose (i.e. duration and intensity) that would influence strength development.

We found a potentially meaningful improvement in upper body muscular endurance corresponding to a moderate effect size, but no meaningful changes in children’s perceived strength or lower body power. Care should be taken when interpreting these findings, given the small sample size and lack of a control group. We cannot determine whether the change in push-ups was simply a learning effect, nor whether the trivial non-significant effects for other outcomes were due to limited statistical power. That said, the push-up was one of the four *Master Moves* taught to and practiced by students, and so improvements in this outcome are plausible. This result is also consistent with a recent meta-analysis of school-based MSA interventions for children, which reported a moderate pooled effect (*g* = 0.65, 95%CI = 0.13 to 1.17) for measures of local muscular endurance and a mean (95%CI) improvement in push-ups of 1.37 (0.91 to 1.83) repetitions among studies using this test [22]. By contrast, powerful jumping movements were not deliberately targeted in *Muscle Movers,* which might explain the trivial effect size for the standing long jump. In the meta-analysis by Villa-Gonzales et al., the pooled effect size for strength/power was half as large as for muscular endurance, and trivial/non-significant among trials using the standing long jump test [22]. Despite being valid/reliable [43], the standing long jump is less appropriate for interventions that do not incorporate plyometric or maximal strength/power training, and an alternative lower body measure such as the 30 s sit-to-stand test may be more suitable. The plank hold test may also be suitable to include in the future, given this also aligns with one of the core skills taught as part of the program. A more robust research design should be used in the future to determine the efficacy of *Muscle Movers* for improving children’s muscular fitness.

### Strengths and limitations

A key strength of our study was the comprehensive, mixed-methods feasibility evaluation conducted with reference to the widely used framework by Bowen and colleagues [36]. In addition, *Muscle Movers* was designed with scalability in mind, and we tested a realistic implementation model that avoids the most common generalisability biases found within prior pilot/feasibility studies of school-based physical activity interventions [49]. However, there are also some important limitations that should be noted. Our sample was small and homogenous, involving only two teachers and their classes from a single school, and we also did not have a control group. These limitations influence the generalisability of our feasibility results to different teachers and school contexts and limit our capacity to determine a causal link between the intervention and the improvement in students’ local muscular endurance. In addition, the trial was not prospectively registered, and a priori progression criteria were therefore not specified. Next, our measure of teachers’ confidence to deliver MSA was adapted from an existing scale, but the adapted measure has not been psychometrically tested with this population, and it is possible validity/reliability differs from the original. In addition, we did not capture data on the volume or intensity of MSA within the PE lessons, which are critical predictors of the strength improvements that might be gained [6]. In a future efficacy trial, it will be important to incorporate a measure of exercise intensity for the purposes of prescription and monitoring. Traditional perceived exertion ratings or more recent scales incorporating repetitions in reserve (e.g. RPE-RIR) might be feasible for this population and setting but require further investigation [57]. Finally, although the intervention aimed to develop students’ MSA-related movement skills (i.e. competence), we did not assess this (or other physical literacy domains), which might be more important at this stage of development than short-term improvements in muscular fitness.

## Conclusions

Our study suggests that the *Muscle Movers* program is feasible for delivery in primary schools, as demonstrated by high teacher and student satisfaction, strong implementation fidelity, and positive teacher perceptions of adaptability and practicality. Taken together, our findings provide justification for conducting an efficacy study of the *Muscle Movers* program using a cluster RCT with a larger and more diverse sample of schools. A future efficacy evaluation of *Muscle Movers* should address areas for further refinement identified by teachers in this study, including the volume of intervention content and strategies to support student engagement with the active homework component. Moreover, a powered cluster RCT will be able to confirm the efficacy of the intervention on students’ muscular fitness and other measures of interest not assessed in this study (e.g. MSA-related movement skill competency).

## Supplementary Information

Additional file 1: Table S1. Core intervention components and description.

Additional file 2: An example lesson plan.

Additional file 3: Flow of participants through the study.

## Data Availability

The datasets used and/or analysed during the current study are available from the corresponding author, J.J.S., on reasonable request.

## Abbreviations

MSA: Muscle-strengthening activity
NSW: New South Wales
PE: Physical education
RT: Resistance training
SD: Standard deviation
