# Supplementary material for: Feasibility of ‘Muscle Movers’: a teacher-delivered program to support children’s participation in muscle-strengthening physical activity
Source: Pilot Feasibility Stud. 2025 Dec 16;12:11. doi: 10.1186/s40814-025-01751-0 (PMC12822025; doi:10.1186/s40814-025-01751-0)
Supplement: Supplementary file 1 — Additional file 1: Table S1. Core intervention components and description. [file 40814_2025_1751_MOESM1_ESM.docx]

**Additional file 1**

**Table 1.** Core intervention components and description

| **PE program** | |
| --- | --- |
| ***Content*** | Week 1 introduced students to all four *Master Moves* (i.e., Scorpion squat, Lemur Lunge, Python plank and Panther Push-up), weeks 2-5 focused on each *Master Move* individually in sequence, and Week 6 aimed to consolidate students’ understanding of all *Master Moves*. PE lessons were separated into three distinct segments: i) *Start Strong*, a simple, brief and enjoyable warm-up activity; ii) *Main Muscles*, the main body of the lesson focused on explicit instruction and application of the allocated *Master Move*; and iii) *Muscle-Up*, an enjoyable and optional extension activity to add further variety for teachers/schools with time available. Teachers were instructed to deliver the *Main Muscles* lesson segment as outlined, but could exchange, repeat, or re-order any *Start Strong* and *Muscle-Up* activities as they wished. This flexibility was provided to support teachers’ autonomy and to address unforeseeable constraints unique to the school/class context (e.g., available space, student motivation/preferences, teachers’ confidence/preferences etc). To address common barriers, PE lesson activities were intended to be simple to organise and explain, engaging for children, and able to be delivered with limited space and equipment. The *Main Muscles* lesson segment used familiar minor games, adapted to incorporate fitness infusion (i.e., the inclusion of vigorous exercise, via the *Master Moves*, in the context of game play). |
| ***Format*** | Delivered outdoors in the school setting, face-to-face, once weekly for 45-60 mins over 6-weeks. |
| ***Facilitator*** | First lesson delivered by member of research team to model correct instruction of *Master Moves*, familiarise teachers with lesson structure and resources, and to build rapport and sense of credibility with teachers. Teachers facilitated program delivery thereafter. |
| ***Pedagogy*** | Perceived competence supported through explicit skill instruction and application of skills in simple games with achievable goals (*Supportive*). Selection of familiar games, requiring limited set-up and explanation to minimise teacher talk/transition time (*Active*). Opportunities for student choice embedded in lesson plans, for example choice of partner for paired tasks (*Autonomous*). Variations to skill execution provided to facilitate differentiation based on skills/fitness (*Fair*). Engaging games/activities selected, music played during lessons through Bluetooth speaker, and variety of games/activities provided (*Enjoyable*). |
| **Energiser breaks** | |
| ***Content*** | *Would you rather* breaks were each based on a distinct theme (e.g., food, occupations etc) and presented students with two options to select from. Students chose which option ‘they’d rather’ and performed a corresponding exercise depicted on screen for 20 seconds (or until volitional failure). *Rock, paper, scissors* breaks were based on the classic game, with students aiming to beat the result displayed on screen. After physically presenting their choice, one of the options was displayed on screen and students determined whether they won, lost or drew the round. They then performed an exercise corresponding with the appropriate outcome for 20 seconds (or until volitional failure). Each energiser included five rounds and took just under five minutes. |
| ***Format*** | Delivered indoors (classroom) in the school setting, face-to-face, supported electronically (PPT slide presentation), twice weekly for 5 minutes over 6 weeks. |
| ***Facilitator*** | Teacher facilitated but supported by electronic resource. Slide transitions were pre-programmed so teacher could simply press play, and then focus on encouraging students, and promoting correct exercise skill execution, rather than modelling all exercises. |
| ***Pedagogy*** | Simple instructions with minimal wait-time, easy for teachers to use (e.g., PPT files with automatic slide transitions) (*Active*). All activities provided opportunities for children to exercise choice or demonstrate preferences (*Autonomous*). Non-competitive activities, participation was achievable irrespective of fitness/skills (*Fair*). Engaging activities that tapped into children’s interests, use of humour and music (*Enjoyable*). |
| **Home tasks** | |
| ***Content*** | Student handbook included six distinct activities designed to engage children and provide opportunity for additional practice of the *Master Moves* outside of the school setting. Examples tasks included: i) guessing the number of repetitions of a selected exercise that could be completed in a given timeframe and comparing guess with result, ii) selecting a song with a repetitive lyric and performing a self-selected exercise each time the lyric appeared when the song was played, iii) teaching a family member how to complete a self-selected *Master Move* by providing a demonstration and giving feedback using the information on the skill card within the handbook, iv) selecting household chores, allocating exercises to each, and completing self-selected repetitions to indicate to caregivers when the chore was completed, v) searching within home reader for words that rhyme with *Master Moves* and completing a repetition of the corresponding exercise when found, and vi) locating facts corresponding to the animals linked to *Master Moves* and completing repetitions after recording the fact in the handbook. |
| ***Format*** | Delivered in the home setting, student self-directed with caregiver support, once weekly for approx. 10 mins over 6 weeks. |
| ***Facilitator*** | Student self-directed, supported by a student handbook with instructions for student and caregivers to refer to. |
| ***Pedagogy*** | Student handbook included *Master Move* skill cards to support competence (*Supportive*). Opportunities for choice embedded within activities (e.g., select a song, choose a book) (*Autonomous*). Activities designed to be engaging for children. Variety provided to avoid boredom (*Enjoyable*). |
