# Supplementary material for: Feasibility of ‘Muscle Movers’: a teacher-delivered program to support children’s participation in muscle-strengthening physical activity
Source: Pilot Feasibility Stud. 2025 Dec 16;12:11. doi: 10.1186/s40814-025-01751-0 (PMC12822025; doi:10.1186/s40814-025-01751-0)

MUSCLE MOVERS

Lesson FIVE

The Panther Push-up

Objectives:

- To develop children's competence in the 'Push-up'
- To apply the 'Push-up' in a game-based movement context
- To provide children with an enjoyable PE experience

Key enquiry questions:

- How can we move our bodies to perform skills in different ways?
- How can we demonstrate our understanding of movement to solve challenges?

Syllabus outcomes: PD2-4, PD2-8, PD2-11

*Lookout also for **Motivators** that are taking responsibility, asking for help, working well with others, or helping their classmates*

THE UNIVERSITY OF NEWCASTLE AUSTRALIA

Heart Foundation

WESTERN SYDNEY UNIVERSITY

Lesson FIVE

Start strong (5-10 mins)

Argy bargy

- Face partner in crouched position, and grab hold of ankles (inside of legs).
- Without lifting backside up, waddle towards partner to ‘barge’ them over.
- Keep hands on ankles and do not barge partner too hard.
- First to lift hands off ankles, stand up, or fall over loses the point.
- Play to 3 points.
- Note. Best delivered on soft surface

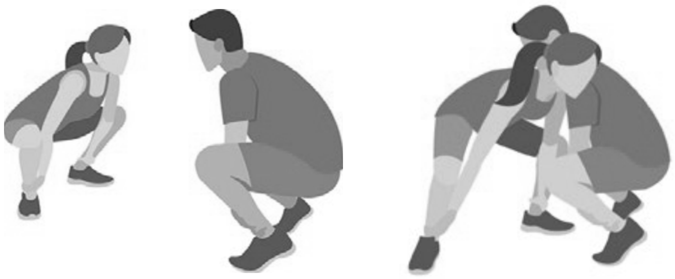

Back-to-back wrestle

- Sit down back-to-back with partner, arms linked and feet flat on ground
- On ‘go’, try to pull partner over to the right-hand side (opposite ways)
- Don’t move feet or use legs for leverage during the ‘wrestle’
- First to pull partner over gets the point.
- Repeat with left side
- Play first to 3 points, alternating sides each time

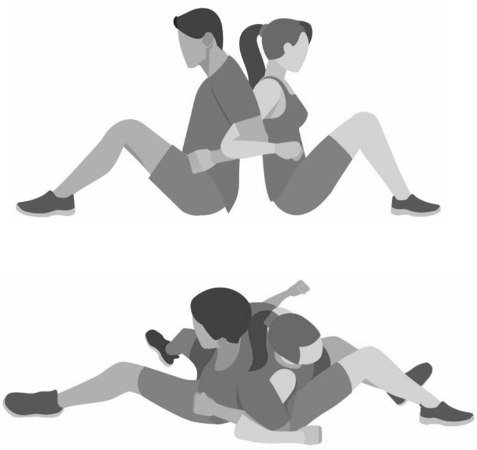

Under the bridge

- One partner begins in plank position (can wait on knees until ready), the other stands next to them
- On ‘go’, child crawls under partner in plank position, and then assumes plank position themselves
- The partner initially in plank then does the same. Repeat for 30 seconds Partners count how many times ‘under the bridge’ they achieve and then try to beat their score in a second round.

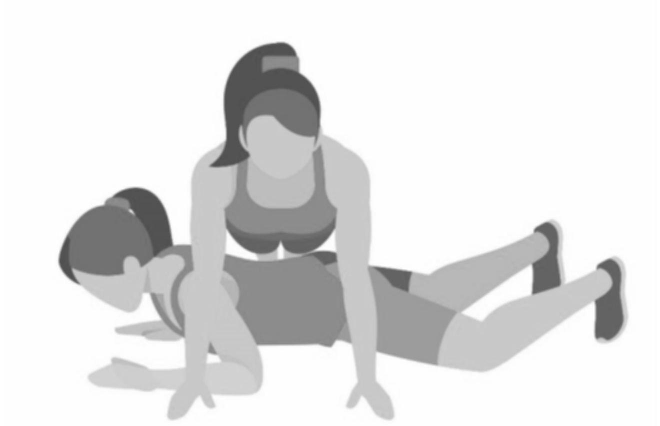

Questioning/reflection:

- What worked well to help you score points in Argy Bargy?
- Who beat their round 1 score in ‘Under the bridge’? What did you do differently the second time to improve your score?

Rate this activity (select one)

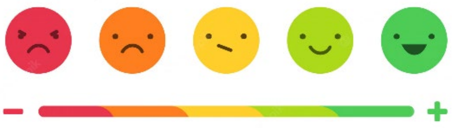

Lesson FIVE

Main Muscles (20-25 mins)

Master Move instruction: Panther Push-up (5-10 mins)

- Check students' recall of how to perform the Panther Push-up (how to 'start', 'move', and 'finish').
- Use Master Move skill cards as a scaffold during explicit teacher instruction of Panther Push-up.
- Class in small groups, each group with a skill card, spaced evenly in front of teacher.
- **Challenge:** Play "baby shark". Class does Push-up every time you hear "shark". Count reps completed.

Skill application: Rob the nest with Panther Push-up (15 mins)

- Separate class into 4 teams, placed at corners of a square marked by cones (about 10 x 10m).
- Place pile of bean bags in middle, and place marker cone between each team and the middle pile (this is the Master Move zone).
- On 'go' ONE student per team moves towards the middle to collect ONE bean bag at a time, and then return it to their team's pile. To start, they must move to first cone and complete a 'belly squash' (Panther Push-up, with belly touching cone), after which they can run to the centre and back.
- Once returned, students 'high five' next group member to move forward and they go to back of line.
- Once all bean bags are gone from middle, students take turns ONE at a time to 'rob the nest' of other teams (only ONE bean bag may be taken at a time!). Teams breaking rules have bean bags taken.
- Nominate time limit from start of 'robbing period' (30 seconds) and count down the final 5 seconds.
- The winning team is the one with the most bean bags (or most points; see progression/variation).

Organisation

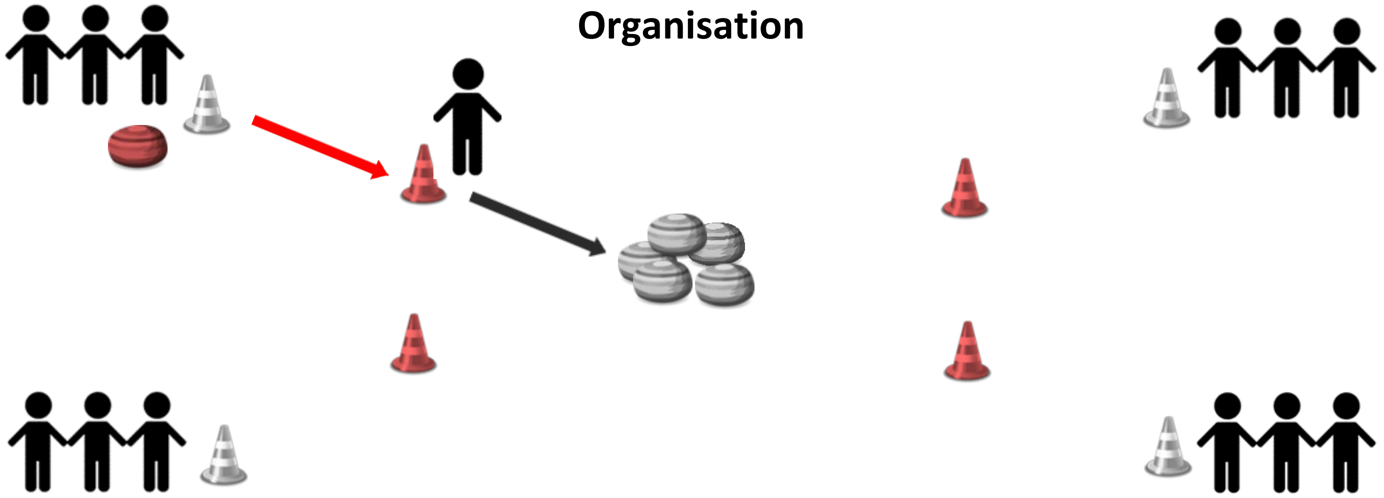

Progressions / variations

- Allocate different point values to different colours and add up at the end, instead of just counting total bean bags.
- Using 'lettered' bean bags, teams compete to make the longest word with their bean bags at the end of the round.

Equipment required:

- Master Move skill cards
- Marker cones
- Bean bags

Questioning/reflection:

- How do we 'start', 'move' and 'finish' the Panther Push-Up?
- What did your team do to try and win rob the nest? Did your strategy work? Why/why not?

Rate this activity (select one)

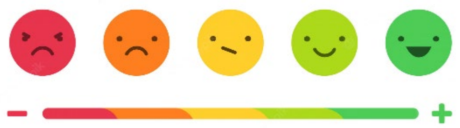

Lesson FIVE

Muscle-up (5-10 mins)

The Monster

- Organise the class into groups of 4 or 5 students (groups should be of equal size where possible)
- Instruct the class that their challenge is to move from the starting line to the finish line 10-20m away.
- They must move together as a group, whilst only ever having 3 points of contact on the ground at one time.
- Groups must try and solve the challenge by communicating and working together as a team.
- There are many ways in which groups could transit the 10-20m distance, including synchronised stepping, lifting group members up etc
- Groups must return to the start if they have more than 3 points of contact on the ground at any time.
- The 'shape' the group makes looks like a monster, made up of several people joined together!

Organisation

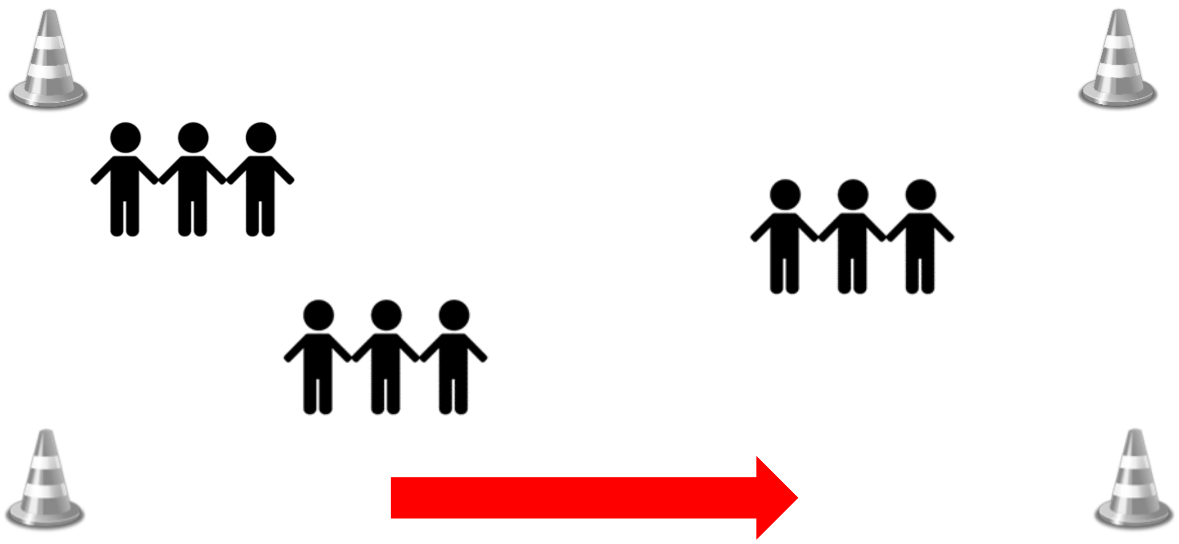

Progressions / variations

- Change the size of the groups, the transit distance, or the number of points of contact allowed to make it easier or harder.
- If groups achieve the challenge, ask them to perform the challenge again using a different strategy
- Reward/recognise the most creative strategy

Equipment required:

- Bluetooth speaker with music
- Marker cones

Questioning/reflection:

- What did you do to help you succeed with this challenge?
- How did you use communication to succeed?

Rate this activity (select one)

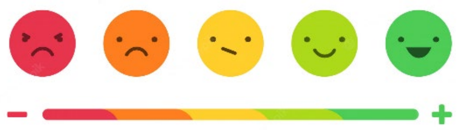

Supplement: Supplementary file 2 — Additional file 2: An example lesson plan. [file 40814_2025_1751_MOESM2_ESM.pdf]
