# Supplementary material for: Feasibility of ‘Muscle Movers’: a teacher-delivered program to support children’s participation in muscle-strengthening physical activity
Source: Pilot Feasibility Stud. 2025 Dec 16;12:11. doi: 10.1186/s40814-025-01751-0 (PMC12822025; doi:10.1186/s40814-025-01751-0)
Supplement: Supplementary file 3 — Additional file 3: Flow of participants through the study. [file 40814_2025_1751_MOESM3_ESM.doc]

**Enrollment**

Assessed for eligibility (n=32)

Excluded (n=2)

♦ Did not return consent form (n=2)

No randomisation

**Allocation**

Allocated to intervention (n=30)

♦ Received allocated intervention (n=30)

**Follow-Up**

Lost to follow-up:

♦ Perceived strength (n=5)

♦ Did not complete survey (n=5)

♦ Push-ups (n=5)

♦ Absent on testing day (n=3)

♦ Precluding injury (n=2)

♦ Standing long jump (n=4)

♦ Absent on testing day (n=3)

♦ Precluding injury (n=1)

Discontinued intervention (give reasons) (n= )

**Analysis**

Analysed (n=25):

♦ Perceived strength (n=25)

♦ Excluded: incomplete data (n=5)

♦ Push-ups (n=25)

♦ Excluded: absent (n=3), injured (n=2)

♦ Standing long jump (n=26)

♦ Excluded: absent (n=3), injured (n=1)

**Additional File 3.** Flow of participants through the study
